# A fungal endophyte helps plants to tolerate root herbivory through changes in gibberellin and jasmonate signaling

**DOI:** 10.1111/nph.13957

**Published:** 2016-04-06

**Authors:** Marco Cosme, Jing Lu, Matthias Erb, Michael Joseph Stout, Philipp Franken, Susanne Wurst

**Affiliations:** ^1^ Functional BiodiversityDahlem Center of Plant SciencesInstitute of BiologyFreie Universität BerlinKönigin‐Luise‐Straße 1–314195BerlinGermany; ^2^ Department of Plant PropagationLeibniz‐Institute of Vegetable and Ornamental CropsKühnhäuser Straße 10199090Erfurt‐KühnhausenGermany; ^3^ Plant–Microbe InteractionsDepartment of BiologyFaculty of ScienceUtrecht UniversityPO Box 800.563508 TBUtrechtthe Netherlands; ^4^ Department of BiochemistryMax Planck Institute for Chemical EcologyHans‐Knoell‐Str. 807745JenaGermany; ^5^ Institute of Insect ScienceZijingang CampusZhejiang UniversityYuhangtang Road 866Hangzhou310058China; ^6^ Institute of Plant SciencesUniversity of BernAltenbergrain 213013BernSwitzerland; ^7^ Department of EntomologyLouisiana State University Agricultural Center404 Life Sciences BuildingBaton RougeLA70803USA; ^8^ Department of Plant PhysiologyHumboldt Universität zu BerlinPhilippstrasse 1310115BerlinGermany

**Keywords:** GA signaling, induced plant tolerance, jasmonate signaling, *Lissorhoptrus oryzophilus* (rice water weevil), *Oryza sativa* (rice), phytohormone crosstalk, *Piriformospora indica* (endophyte), root herbivory

## Abstract

- Plant–microbe mutualisms can improve plant defense, but the impact of root endophytes on below‐ground herbivore interactions remains unknown. We investigated the effects of the root endophyte *Piriformospora indica* on interactions between rice (*Oryza sativa*) plants and its root herbivore rice water weevil (RWW;* Lissorhoptrus oryzophilus*), and how plant jasmonic acid (JA) and GA regulate this tripartite interaction.
- Glasshouse experiments with wild‐type rice and *coi1‐18* and *Eui1‐OX* mutants combined with nutrient, jasmonate and gene expression analyses were used to test: whether RWW adult herbivory above ground influences subsequent damage caused by larval herbivory below ground; whether *P. indica* protects plants against RWW; and whether GA and JA signaling mediate these interactions.
- The endophyte induced plant tolerance to root herbivory. RWW adults and larvae acted synergistically via JA signaling to reduce root growth, while endophyte‐elicited GA biosynthesis suppressed the herbivore‐induced JA in roots and recovered plant growth.
- Our study shows for the first time the impact of a root endophyte on plant defense against below‐ground herbivores, adds to growing evidence that induced tolerance may be an important root defense, and implicates GA as a signal component of inducible plant tolerance against biotic stress.

## Introduction

Plants require sophisticated defense mechanisms supported by microbial alliances to defend themselves against a broad spectrum of heterotrophic attackers, including insect herbivores (Rodriguez *et al*., 2009; Erb *et al*., 2012b; Pieterse *et al*., 2014). Herbivores attack from above and below ground, and both shoots and roots deploy resistance mechanisms that reduce herbivore infestation and performance (Howe & Jander, 2008; Lu *et al*., 2015) as well as tolerance mechanisms that allow regrowth and fitness recovery after tissue damage (Strauss & Agrawal, 1999; Poveda *et al*., 2010; Robert *et al*., 2014). Compared with the well‐documented role of microbes in induced plant resistance above ground (Hartley & Gange, 2009; Rodriguez *et al*., 2009; Pieterse *et al*., 2014), little is known about the mechanisms that govern microbe‐mediated plant defense against below‐ground herbivores.

Plants allocate a large part of their primary production to below‐ground tissues, where insect herbivores of at least 25 families feed, including many critical agricultural pests (Hunter, 2001; Erb *et al*., 2012a). Losses of plant productivity caused by root herbivory can be amplified when combined with above‐ground herbivory (Zvereva & Kozlov, 2012). Combined shoot and root injury is a common scenario for many insect species whose adults feed on leaves and whose larvae feed on roots (Clark *et al*., 2011; Cosme *et al*., 2011; Currie *et al*., 2011).

The perception of above‐ground chewing herbivory by plants triggers a sophisticated defensive machinery with jasmonic acid (JA) as the central signal (Howe & Jander, 2008). JA can also reduce shoot growth via antagonistic interaction with the GA signaling pathway (Yang *et al*., 2012; Heinrich *et al*., 2013; Matschi *et al*., 2015). Regulation of GA biosynthesis and interactions between DELLA and JAZ proteins are central to JA and GA signaling crosstalk which ultimately modulates growth–defense tradeoff in shoots. Several lines of evidence suggest that JA may regulate root resistance to below‐ground herbivores: root herbivory induces JA signaling in roots (Lu *et al*., 2015); exogenous application of jasmonates reduces root herbivore infestation and survival (McConn *et al*., 1997; Omer *et al*., 2000; Hamm *et al*., 2010; Lu *et al*., 2015); and JA‐deficient rice plants may suffer greater root damage from below‐ground herbivory (Lu *et al*., 2015). However, roots commonly display a much weaker herbivore‐induced JA burst than leaves and other plant signals might be more important for induced defenses to below‐ground herbivores (Erb *et al*., 2012a; Acosta *et al*., 2013). Jasmonates can also reduce root growth, both locally and systemically within the root system, as demonstrated by exogenous application of methyl jasmonate (MeJA) (Staswick *et al*., 1992; Moons *et al*., 1997; Lu *et al*., 2015), whereas GA promotes root growth by controlling cell elongation and root meristem size (Ubeda‐Tomás *et al*., 2009). Whether JA and GA signaling crosstalk regulates regrowth as a tolerance mechanism against root herbivores remains to be determined.

Plant signaling pathways are also modulated by nonpathogenic microbes that colonize roots without producing disease symptoms in the plant. Arbuscular mycorrhizal fungi (AMF), for instance, interact with nearly all known phytohormone pathways (Gutjahr, 2014; Pozo *et al*., 2015). Many studies have shown that AMF can affect the interactions of plants with above‐ground herbivores (Hartley & Gange, 2009; Koricheva *et al*., 2009; Pineda *et al*., 2013), and a role for the JA pathway in AMF‐mediated resistance to foliar‐feeding insects has recently been demonstrated (Song *et al*., 2013). By comparison, the effects of AMF on root‐feeding insects are less well understood (Gange, 2001; Currie *et al*., 2011; Jung *et al*., 2012). Among the many nonpathogenic microbes that colonize roots without forming mycorrhizal structures, *Piriformospora indica* (Sebacinales) stands out because of its exceptionally broad host range and its positive effects on plant productivity via increases in plant tolerance to abiotic stresses and resistance to pathogens (Varma *et al*., 1999; Barazani *et al*., 2005; Waller *et al*., 2005; Qiang *et al*., 2012). *P. indica* manipulates particular branches of the signaling network of its host plants. For instance, it requires JA signaling in roots during biotrophic root colonization, while during cell death‐associated colonization the endophyte recruits GA signaling to degrade DELLAs and establish cell apoptosis susceptibility (Schäfer *et al*., 2009; Jacobs *et al*., 2011). To date, the impact of root endophytes on below‐ground herbivore interactions remains unknown.

Here, we investigated the effects of *P. indica* on rice (*Oryza sativa*) defense against a major root pest, the rice water weevil (RWW; *Lissorhoptrus oryzophilus*). The RWW is native to North America but is now present in rice paddies around the globe (Stout *et al*., 2013). The adults feed on leaves without causing significant damage, but the root‐feeding larvae markedly reduce rice productivity (Stout *et al*., 2002; Zou *et al*., 2004). Using this system, we tested the hypotheses that: (H1) above‐ground feeding by RWW adults on leaves enhances subsequent damage caused below ground by conspecific root‐feeding larvae; (H2) prior root colonization by *P. indica* protects rice plants against RWW attack; and (H3) JA and GA signaling pathways in rice mediate this tripartite interaction.

## Materials and Methods

### Plant, fungus, insect and soil

Wild‐type (WT) rice (*Oryza sativa* L., cultivar Nipponbare) was used as the background of all plant mutants. In experiment I (Expt I), we used WT seeds kindly provided by Dr Claus‐Peter Witte (Freie Universität Berlin, Germany). In Expt II, we used seeds of WT, *coi1‐18* and *Eui1‐OX* kindly provided by Prof. Dr Zuhua He (Chinese Academy of Sciences, China). The coronatine insensitive 1 (COI1) protein is a principal component of the JA receptor complex in rice and other plants. As a consequence, silencing *COI1* reduces responsiveness to JA (Yang *et al*., 2012). The elongated uppermost internode 1 (EUI1) protein is involved in GA catabolism in rice (Luo *et al*., 2006; Zhu *et al*., 2006; Zhang *et al*., 2008; Yang *et al*., 2012). Overexpressing *EUI1* (*Eui1‐OX*) reduces GA concentrations and results in dwarfed plants. All plants used in the present experiments were germinated on Murashige and Skoog medium in Petri dishes over 3 d and planted according to the experimental designs.

The fungal root endophyte *Piriformospora indica* Varma, Rexer, Kost & Franken (Sebacinales, Basidiomycota; strain DSM 11827 in Deutsche Sammlung für Mikroorganismen und Zellkulturen, Braunschweig, Germany) is a rare species isolated from the Indian Thar desert (Verma *et al*., 1998). *P. indica* was propagated at the Leibniz Institute of Vegetable and Ornamental Crops (Großbeeren, Germany) by routine procedures on potato dextrose agar (PDA) in Petri dishes for Expt I or in liquid culture containing a complete medium for Expt II (Verma *et al*., 1998).

Adults of the RWW (*Lissorhoptrus oryzophilus* Kuschel, Coleoptera: Curculionidae) were collected from flooded rice fields at the Louisiana State University Agricultural Center Rice Research Station (Louisiana, USA) and maintained in a laboratory as previously described (Cosme *et al*., 2011). RWW adults were captured *in copula* and used in the leaf infestation bioassays. RWW neonates were reared *in vivo* using freshly germinated rice seedlings and were used in the root infestation bioassays (Zhang *et al*., 2004).

A sandy loam soil from Berlin (52°28′N, 13°18′E) was sieved and mixed with peat (Floragard Vertriebs GmbH, Oldendurg, Germany) and sand (CEMEX GmbH, Kraatz, Germany) to produce the soil substrate (2 : 1 : 1, v/v/v). The soil substrate was fertilized in the pots with 125 ml of 0.05% solution of GABI Plus 12‐8‐11 N–P–K fertilizer (Detia Freyberg GmbH, Laudenbach, Germany) L^–1^ soil substrate.

### Expt I: design and growth conditions

To test the hypothesis H1 that above‐ground feeding by RWW adults on leaves intensifies subsequent damage caused by conspecific root‐feeding larvae below ground, we conducted a full factorial experiment in a glasshouse (16 : 8 h, 28 : 22°C, day : night). The roots of 3‐d‐old WT rice seedlings were dipped overnight in a sterile 0.05% Tween‐20 aqueous solution to establish the control for *P. indica* inoculation (described later) (*n *=* *32). Each seedling was then planted into 16 × 16 cm round Teku pots filled with 2 l of soil substrate. To confine the roots and larvae within the pot, a Plantex DuPont mesh had been previously glued with silicone onto the bottom of each pot. The soil substrate was regularly moistened with tap water. At 15 d after germination (DAG), a 7 × 5 cm round clip cage was attached to the first leaf with a mating pair of RWW adults inside (*n *=* *16). An identical clip cage without adults (*n *=* *16) was clipped to uninfested control plants. All plants infested with RWW adults showed numerous leaf feeding scars 2 d after infestation. This time period is within standard protocols for adult feeding bioassays (Stout *et al*., 2002; Lupi *et al*., 2013). The adults were then re‐collected, the clip cage was removed, and a photograph of the injured leaf was recorded using a standardized focal distance. Control plants were photographed under the same conditions. The photos were analyzed using WinDIAS 3.1 software (Delta‐T Devices, Cambridge, UK) to determine the leaf area consumed by RWW adults. In the field, the flooding of rice paddies typically takes place a few weeks after seeding (Stout *et al*., 2002). The presence of standing water then triggers RWW oviposition and females lay eggs in submerged rice leaf sheaths (Stout *et al*., 2002). The eggs hatch 4–9 d after oviposition and the neonates mine the leaf tissue for a short time but eventually move to the roots where they develop through four instars until adulthood (Cosme *et al*., 2011; Aghae & Godfrey, 2014). To simulate the life history of RWW in our experiment, the pots were placed into 24 × 20 cm round plastic buckets and flooded with tap water at 23 DAG. Thirty‐six days after germination, the plants with adult feeding scars (*n *=* *8) and their corresponding controls without scars (*n *=* *8) received eight neonates per plant over 4 d to establish the root infestation treatment. Infestations of eight or more neonates per plant is common in the field (Stout *et al*., 2013). The remaining plants were not infested with larvae as controls (*n *=* *8 + 8).

To test hypothesis H2 that *P. indica* can protect rice plants against RWW attack, we simultaneously conducted the same RWW treatments described earlier on rice plants previously inoculated with *P. indica* (*n *=* *32). The inoculation was established by dipping the roots of 3‐d‐old WT rice seedlings overnight in a 0.05% Tween‐20 aqueous solution containing 4.9 × 10^7^ ml^−1^ chlamydospores of *P. indica*. This concentration of chlamydospores was chosen because it leads to good colonization of rice roots (P. Franken, unpublished). The RWW larvae develop through four instars in 21–27 d before forming pupae (Hamm *et al*., 2010). To provide maximal exposure of larvae to roots and avoid eclosion, rice plants were harvested 22 d after neonate infestation (58 DAG). All experimental plants (*n *=* *64) were treated and distributed in randomized fashion on a glasshouse table.

### Expt I: plant, larval and fungal performance

To determine the growth of plants from Expt I described earlier, shoots and roots were excised separately at 58 DAG. The number of tillers for each plant was counted and the soil substrate was carefully washed from roots. Subsamples of the youngest leaf and of intact root tissue without visible symptoms of wounding were immediately frozen in liquid nitrogen and stored at −80°C to analyze jasmonate concentrations and gene expression as described later. To recover larvae 22 d after neonate infestation, the soil substrate and roots were screened carefully in buckets filled with water. The larvae and pupae were counted as they floated to the surface (Zou *et al*., 2004). The FW of insects and plants were measured. The total root length and average root diameter for each plant were determined using WinRhizo software (Regent Instruments Inc., Québec, Canada) as previously described (Cosme & Wurst, 2013). To quantify endophyte colonization 55 d after inoculation, subsamples of root fragments were stained using trypan blue solution and then destained before observation with a microscope (Phillips & Hayman, 1970). The percentage of root length colonized by the endophyte was quantified as the percentage of microscope fields of view containing root segments with chlamydospores as adapted from McGonigle *et al*. (1990).

### Expt I: mineral elements in shoot

After measuring plant biomass, the shoots of the 58‐d‐old rice plants were dried in an oven (60°C for 1 wk) and homogenized into a fine powder using sintered corundum alumina jars and balls in a Planetary Micro Mill Pulverisette 7 (Fritsch, Idar‐Oberstein, Germany). To assess the mineral nutrition status of rice plants, the concentrations of nitrogen (N), phosphorus (P), potassium (K), calcium (Ca), magnesium (Mg), sulfur (S), manganese (Mn), iron (Fe), zinc (Zn), boron (B), copper (Cu) and molybdenum (Mo) in shoots were measured using a CN Elemental Analyzer (Euro EA, HEKAtech GmbH, Wegberg, Germany) or an inductively coupled plasma‐optical emission spectrometer (iCAP ICP‐OES Duo; Thermo Fisher Scientific Inc., Waltham, MA, USA) as previously described (Cosme *et al*., 2014).

### Expt I: jasmonates in leaves and roots

To quantify the concentrations of jasmonates in 58‐d‐old rice plants (i.e. 55 d after inoculation with *P. indica*, 43 d after leaf infestation with RWW adults and 22 d after root infestation with RWW neonates), 12‐oxophytodienoic acid (OPDA), JA and jasmonoyl‐isoleucine (JA‐Ile) were extracted from frozen leaf and root subsamples (*n *=* *8) following Lu *et al*. (2015). The extracts were analyzed by LC‐MS using an API 3200^™^ LC/MS/MS system (Applied Biosystems, Framingham, MA, USA) as previously described (Vadassery *et al*., 2012).

### Expt I: gene expression in roots

Quantitative real‐time PCR (qRT‐PCR) analyses were conducted following Lu *et al*. (2015). To assess *de novo* biosynthesis in JA and GA signaling pathways, we analyzed the gene expression of JA‐Ile synthase *OsJAR1* (Riemann *et al*., 2008) and *ent*‐Kaurene synthase *OsKS1* (Sakamoto *et al*., 2004) (Supporting Information Table S1), respectively. To normalize cDNA concentrations, we used the rice actin *OsACT* as a housekeeping gene (Table S1). qRT‐PCR was performed with Mx3000P qPCR System (Stratagene, La Jolla, CA, USA) using Brilliant III Ultra‐Fast SYBR^®^ Green QPCR Master Mix (Agilent Technologies, Santa Clara, CA, USA). The relative expression levels of genes were calculated using the double standard curve method.

### Expt II: design and growth conditions

**Figure 1:**
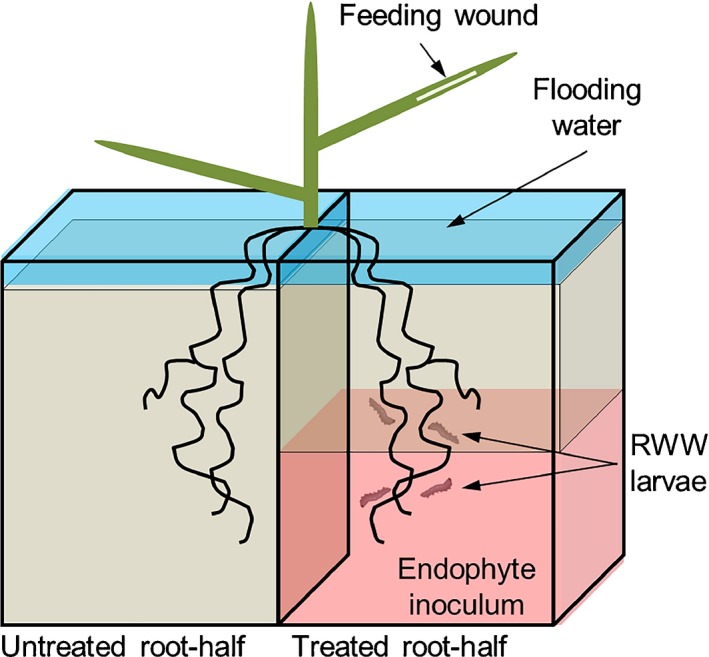
Schematic representation of a split‐root system. The split‐root system consisted of two hydroponic square pots paired side by side, with the root system equally divided into two halves, where one root‐half received soil treatments, that is, endophyte *Piriformospora indica* and/or larvae of rice water weevil (RWW;* Lissorhoptrus oryzophilus*), and the other root‐half was left untreated. The endophyte inoculation was added only to one side by filling half of the hydroponic pot with a soil previously mixed with endophyte mycelium. Uninoculated plants had a similarly pot side half filled with soil previously mixed with autoclaved mycelium.

To test whether RWW larvae reduce root growth locally or systemically within roots, we repeated the RWW infestation treatments applied in Expt I (described earlier) with minor alterations adapted to a split‐root system (Fig. 1), in which only one‐half of the root system was treated with RWW larvae. Hydroponic 1 l square pots were paired by gluing two pots side by side. Each pot was filled with 500 ml of soil substrate before transplanting. One side of the split‐root system was assigned to either an RWW larval infestation (*n *=* *16) or an uninfested control (*n *=* *16) treatment, and this side of the split‐root received a mock inoculum of autoclaved (121°C for 20 min) endophyte mycelium and spores (4 mg) to establish the control for endophyte inoculation (described later) (*n *=* *32). To allow enough root growth before transplanting, 3‐d‐old WT rice seedlings were planted in nursery trays filled with 60 ml of soil substrate per vessel. Rice roots were carefully washed 20 DAG and transplanted into the split‐root system by dividing the roots into two halves. Each pot side of the split‐root systems was then filled with 500 ml additional soil substrate to completely cover the root system, resulting in 2 l of soil subtract for each plant. At 26 DAG, a clip cage was attached to the first leaf with a mating couple of RWW inside (*n *=* *16). An identical clip cage without adults was attached to control plants (*n *=* *16). All plants infested with RWW adults showed leaf‐feeding scars 2 d after infestation. The split‐root systems were flooded with tap water at 28 DAG. The plants with adult feeding scars (*n *=* *8) and their controls (*n *=* *8) received 16 RWW neonates in one‐half of the root system at 30 DAG. We doubled the number of neonates relative to Expt I to inflict a potentially higher amount of local injury in this experiment. Infestation densities were well within the range of densities observed in the field (Stout *et al*., 2013). The remaining plants were kept uninfested as controls (*n *=* *8 + 8).

To test whether the endophyte inhibits the systemic effects of RWW larvae on root growth, we simultaneously conducted the same RWW treatments in the split‐root system described earlier using plants previously inoculated with the endophyte (*n *=* *32). For this purpose, *P. indica* was cultivated and harvested following Fakhro *et al*. (2010), with the exception that a complete medium (Pontecorvo *et al*., 1953) was used instead of potato dextrose broth. The inoculation was established by mixing 4 mg of endophyte mycelium and spores into the pot‐side assigned to RWW larval treatment (*n *=* *16) or one pot‐side in the uninfested larval control treatment (*n *=* *16) before transplanting the rice plants. To test whether JA signaling mediates the RWW effects on plant growth, we employed the same experimental treatments simultaneously in split‐root systems using JA‐insensitive *coi1‐18* rice mutant as the host plant (Yang *et al*., 2012). To test whether the endophyte suppression of the effects of RWW on rice requires GA signaling, we simultaneously employed the same experimental treatments in the split‐root system using the GA‐deficient *Eui1‐OX* rice mutant as the host plant (Zhu *et al*., 2006). All experimental plants (*n *=* *192) were treated and distributed in randomized fashion in a glasshouse (14 : 10 h, 28 : 24°C, day : night) and were harvested 28 d after neonate infestation (58 DAG).

### Expt II: plant, larval and fungal performance

We analyzed the same performance parameters described in Expt I with the exception of root morphology and leaf area consumed. The root halves from the split‐root system were excised and analyzed separately.

### Data analysis

Statistical analyses were performed in R Studio Desktop software (http://www.rstudio.com/). All data on plant responses were analyzed by factorial three‐way ANOVAs with the two‐level factors ‘endophyte’ (−, +), ‘RWW adults’ (−, +) and ‘RWW larvae’ (−, +). Leaf area consumed by RWW adults was analyzed by one‐way ANOVA with the two‐level factor ‘endophyte’. Endophyte root colonization was analyzed by two‐way ANOVA with the two‐level factors ‘RWW adults’ and ‘RWW larvae’. Data on larval performance were analyzed by two‐way ANOVA with the two‐level factors ‘endophyte’ and ‘RWW adults’. We checked the assumptions of ANOVA (using Shapiro and Levene tests), and data were transformed if necessary using the appropriate transformations, specifically log, arcsine and square‐root transformations. When transformation did not meet assumptions, or when sample sizes differed, we performed ANOVA using a generalized linear model with best‐fit family errors. *P‐*values between 0.10 and 0.05 were considered trends.

## Results

### Experiment I

Plants of WT rice were inoculated with *P. indica* at 3 DAG, subjected during 2 d to leaf herbivory by RWW adults 15 DAG, and finally infested with root‐feeding RWW larvae at 36 DAG, in a fully crossed glasshouse experiment. Plants were harvested at 58 DAG to determine plant, larval and fungal performance, mineral elements in shoot, jasmonates in leaves and roots, and gene expression in roots.

#### *Piriformospora indica* restores growth of herbivore attacked plants

**Figure 2:**
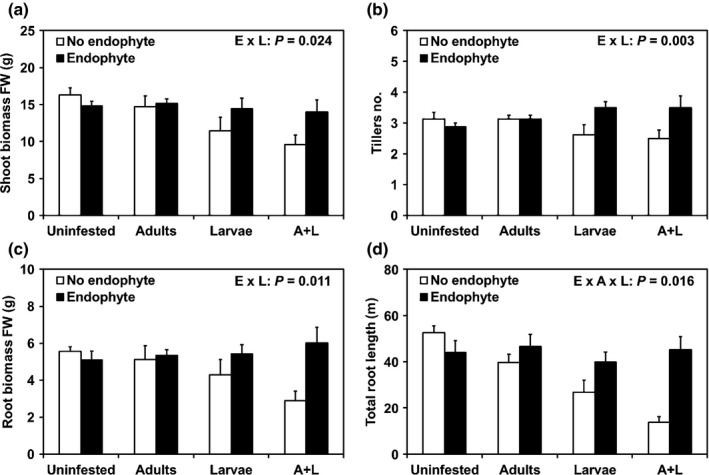
*Piriformospora indica* restores growth of herbivore‐attacked plants. Rice plants (*Oryza sativa* cv Nipponbare) were inoculated with the fungal root ‘endophyte’ *P. indica* 3 d after germination (DAG), then infested above ground on the first leaf with rice water weevil (RWW;* Lissorhoptrus oryzophilus*) ‘adults’ at 15 DAG, and finally infested below ground with RWW ‘larvae’ at 36 DAG, in a fully crossed experiment in glasshouse. The shoot biomass (a), number of tillers (b), root biomass (c), and total root length (d) were measured at 58 DAG and their values were analyzed by three‐way ANOVA. Data are means ± SE (*n *=* *8). Significant *P*‐values of the higher‐order interactions are shown (E, endophyte; A, RWW adults; L, RWW larvae). For other *P*‐values see Supporting Information Table S2.

The growth and root morphology of the 58‐d‐old rice plants were evaluated to test the hypotheses that above‐ground feeding by RWW adults on leaves enhances subsequent damage caused by conspecific root‐feeding larvae below ground (H1), and that prior root inoculation with *P. indica* protects rice plants against RWW attack (H2). RWW adults alone neither affected nor interacted with other factors to affect shoot and root biomasses or numbers of tillers. However, we detected significant interactions between the effects of the endophyte and the larvae on these plant parameters (Table S2). As expected, considerable damage in endophyte‐free plants infested with RWW larvae was observed, that is, endophyte‐free plants infested with RWW larvae produced 32% less shoot biomass (Fig. 2a), 18% fewer tillers (Fig. 2b), and 32% less root biomass (Fig. 2c) than did endophyte‐free, uninfested plants. The most remarkable result was probably the protective impact of the endophyte on the growth of plants infested with RWW larvae. Endophyte‐inoculated plants infested with RWW larvae produced 26% more shoot biomass (Fig. 2a), 27% more tillers (Fig. 2b), and 36% more root biomass (Fig. 2c) compared with endophyte‐free plants infested with RWW larvae, whereas no differences were observed between endophyte‐free plants without larvae and the endophyte‐inoculated plants with or without larvae (Fig. 2a–c). In addition, we found a significant three‐way interaction between the endophyte, the RWW adults and the larvae on total root length (Table S2). Feeding by RWW adults enhanced the negative effect of their conspecific larvae on total root length in endophyte‐free plants (Fig. 2d), with both types of injury combined causing a 74% decrease in length compared with endophyte‐free, uninfested plants. However, the endophyte almost completely suppressed this synergistic negative effect of RWW adults and larvae (Fig. 2d). By contrast, the average root diameter was 7% larger (Table S2) in plants infested with RWW larvae (0.281 ± 0.003 mm) than in uninfested plants (0.263 ± 0.004 mm). The average root diameter was not affected by the endophyte or the RWW adults, and no interactions between factors were detected (Table S2). Taken together, these results indicate that the RWW adults exacerbated the negative effects of the larvae on root development, while the endophyte protected rice plants against the negative effects of RWW injury.

#### Rice nutritional deficit caused by root herbivory is not affected by *P. indica*

We determined the concentrations of 12 macro‐ and micronutrients in rice shoots to assess whether RWW caused plant nutritional deficits and whether these deficits were affected by the endophyte. Root‐feeding by RWW larvae was found to reduce the concentrations of P, K and Mn in shoots by 16%, 6% and 12%, respectively, compared with uninfested plants (Table S3). Despite root pruning by larval feeding, we discovered that plants infested with RWW larvae accumulated 17% more Ca and B and we observed a trend suggesting slightly more S in shoots compared with uninfested plants (Table S3). Furthermore, colonization by the endophyte led to a 7% reduction of Ca in shoots and a small but significant 5% increase of P in shoots compared with that of endophyte‐free plants, and interacted with RWW larvae to suppress the larvae‐mediated increase of Mo in shoots (Table S3). The RWW adults did not affect nutrient accumulation in shoots and no other interactions between factors were detected (Table S3). The concentrations of N, Mg, Zn, Fe, and Cu in shoots were unaltered by the treatments in this experiment (Table S3). Overall, the results suggest that the few nutritional deficits caused by root herbivory in rice were not changed by the RWW adults or the endophyte.

#### *Piriformospora indica* induces GA biosynthesis and suppresses herbivore‐induced JA in roots

**Figure 3:**
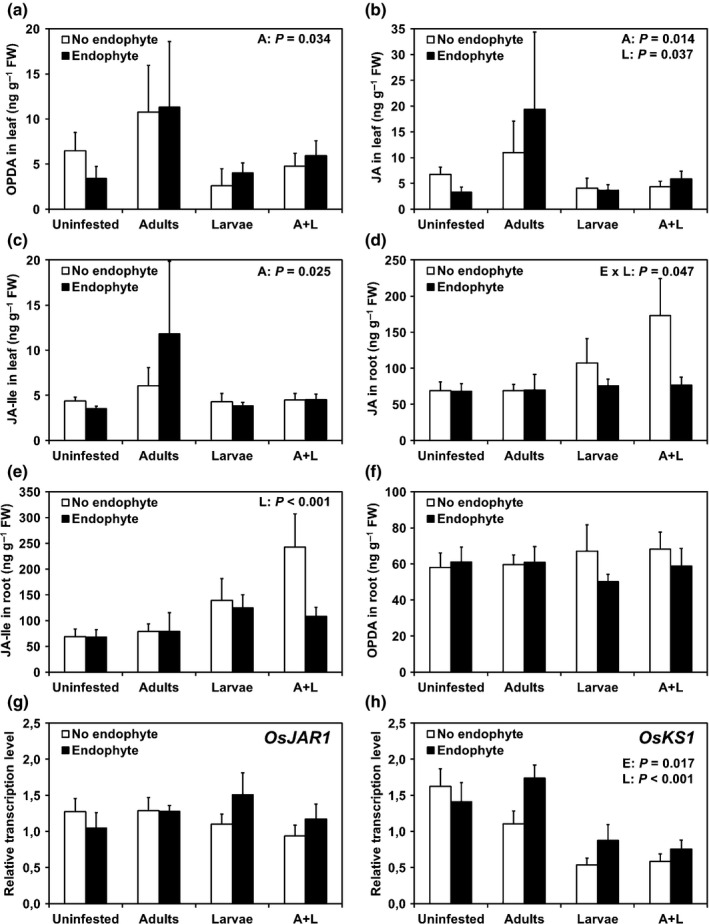
*Piriformospora indica* induces a synthase gene of the GA pathway and suppresses herbivore‐induced jasmonic acid (JA) in roots. Rice plants (*Oryza sativa* cv Nipponbare) were inoculated with the fungal root ‘endophyte’ *P. indica* 3 d after germination (DAG), then infested above ground on the first leaf with rice water weevil (RWW;* Lissorhoptrus oryzophilus*) ‘adults’ at 15 DAG, and finally infested below ground with RWW ‘larvae’ at 36 DAG, in a fully crossed experiment in glasshouse. The concentration of 12‐oxophytodienoic acid (OPDA), JA, and jasmonoyl‐isoleucine (JA‐Ile) in leaves (a–c) and roots (d–f) were measured. The relative transcription abundances in roots of the JA‐Ile synthase (g) *OsJAR1* gene and the *ent*‐Kaurene synthase (h) *OsKS1* gene of the GA pathway were determined. All values were analyzed by three‐way ANOVA using a generalized linear model. Data are means ± SE: (a–c) *n *=* *8; and (d–h) *n *=* *7–8. Significant *P*‐values of the higher‐order interaction or the main factor effects are shown (E, endophyte; A, RWW adults; L, RWW larvae). For other *P*‐values, see Supporting Information Tables S4 (a–f) and S5 (g, h).

To profile plant JA signaling responses to above‐ and below‐ground herbivory under endophyte colonization, we quantified the amounts of OPDA, JA and JA‐Ile in leaves and roots of rice. Plants infested 15 DAG for 2 d with RWW adults produced 99% more OPDA, 130% more JA and 68% more JA‐Ile in leaves at the end of the experiment than did uninfested plants (Fig. 3a–c). Furthermore, we found that leaves of plants infested with RWW larvae had 56% less JA, and observed two trends suggesting less OPDA and JA‐Ile, compared with uninfested plants (Fig. 3a–c). Moreover, OPDA, JA and JA‐Ile in leaves were not affected by the endophyte, and no interactions between factors were detected. In roots, we detected an interaction between the effects of the endophyte and larvae on JA accumulation (Fig. 3d). Endophyte‐free plants infested with RWW larvae accumulated 52% more JA in roots compared with uninfested plants, but when plants were inoculated with the endophyte, the larvae‐mediated accumulation of JA was suppressed (Fig. 3d). Moreover, RWW larvae induced concentrations of JA‐Ile in roots (Fig. 3e) and we observed a trend suggesting that the endophyte may have caused a reduction (Fig. 3e; Table S4). Although RWW adults did not significantly affect concentrations of JA and JA‐Ile in roots, they apparently enhanced the induction by their conspecific larvae, leading to the highest concentrations of JA and JA‐Ile (Fig. 3d,e). The concentration of OPDA in roots was not affected by RWW larvae or the endophyte and no interactions between factors were detected (Fig. 3f; Table S4).

To assess *de novo* biosynthesis in JA and GA signaling pathways, we analyzed gene expression of *OsJAR1* and *OsKS1* in rice roots. The transcription abundance of *OsJAR1* was not significantly affected by the endophyte, the RWW adults or the larvae, and no interactions between these factors were detected. However, we observed a trend for an interaction between endophyte and larvae, suggesting that colonization by the endophyte may have counteracted a minor reduction in *OsJAR1* transcripts following larval infestation (Fig. 3g; Table S5). The endophyte alone elicited 20% more transcription of *OsKS1* in roots compared with that of endophyte‐free plants (Fig. 3h). By contrast, the transcription abundance of *OsKS1* was found to be 54% lower in roots infested with RWW larvae than in uninfested roots (Fig. 3h). Furthermore, we observed a trend for an interaction among the endophyte, RWW adults, and RWW larvae on the transcription abundance of *OsKS1* in roots (Fig. 3h; Table S5). This trend suggests that endophyte‐inoculated plants infested with RWW adults or larvae, or both, had slightly higher transcription abundances of *OsKS1* in roots compared with their respective endophyte‐free control plants, but this difference was slightly clearer in plants infested solely with RWW adults. Altogether, the results suggest that *P. indica* induces *de novo* biosynthesis of components of the GA pathway and indicate that, although *de novo* biosynthesis in the JA signaling pathway was not observed at harvest, the larvae‐mediated accumulation of JA in roots was suppressed by *P. indica*.

#### *Piriformospora indica* does not affect plant resistance against RWW

The leaf area consumed by RWW adults after 2 d of bioassay in clip cages was not altered by the presence of the root endophyte and was 0.244 ± 0.019 cm^2^ (mean ± SE, *n *=* *32). Furthermore, survival of RWW larvae was not altered by the endophyte or by RWW adult feeding, and no interaction between the two factors was detected. Survival of RWW larvae was 46.88 ± 3.68% (*n *=* *32). Likewise, the average weight of RWW larvae and pupae was 17.16 ± 1.31 mg (*n *=* *32) and was not affected by the endophyte or by RWW adult feeding, and no interaction between these factors was detected. Taken together, these results indicate that the fungal endophyte did not affect the leaf and root resistance against RWW in this experiment.

#### Root herbivory decreases endophyte sporulation

Infestation of rice plants with RWW larvae reduced the percentage of root length colonized by the endophyte chlamydospores 55 d after fungal inoculation, from 43.19 ± 5.99% in uninfested plants to 26.69 ± 5.21% in plants infested for 22 d (ANOVA, *F*_1,28_ **= 4.4, *P *=* *0.046; mean ± SE; *n *=* *16). It should be noted that only the numbers of chlamydospores were quantified, because *P. indica* forms very thin hyphae that are difficult to observe. It was therefore not possible to determine whether overall fungal development or only chlamydospore production was affected by the presence of the RWW larvae. The 2 d infestation with RWW adults in 15‐d‐old rice plants did not change the chlamydospore colonization at the end of the experiment, and no interaction between adult and larval infestations was detected. No chlamydospore colonization was detected in any of the 32 control plants treated with the sterile mock inoculum.

### Experiment II

Twenty‐day‐old rice plants of WT, *coi1‐18* and *Eui1‐OX* lines were transplanted into split‐root systems, in which only one‐half of the root system was subject to below‐ground treatments. The plants were inoculated with *P. indica* at transplantation in one root‐half, then subjected during 2 d to leaf herbivory by RWW adults at 26 DAG, and finally infested with RWW larvae in the same root‐half at 30 DAG, in fully crossed glasshouse experiments. Plants were harvested at 58 DAG to determine plant, larval and fungal performance.

#### Endophyte and herbivore have dissimilar effects on systemic plant growth responses

**Figure 4:**
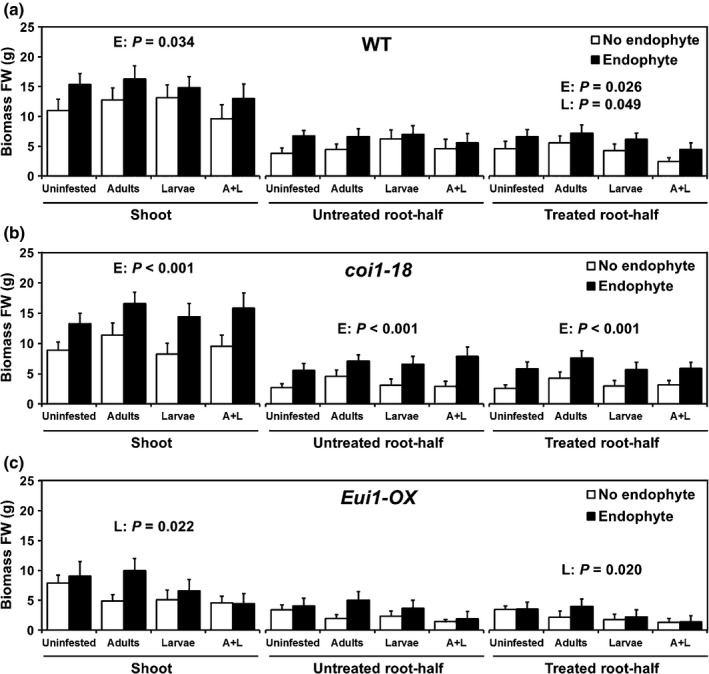
*Piriformospora indica* and rice water weevil (RWW) have dissimilar effects on systemic plant growth responses, and jasmonic acid and GA antagonize these effects. The rice (*Oryza sativa* cv Nipponbare) wild‐type (WT), jasmonic acid (JA)‐insensitive *coi1‐18*, or GA‐deficient *Eui1‐OX* mutant were planted 20 d after germination (DAG) in a split‐root system where only one‐half of the root was treated with the fungal root ‘endophyte’ *P. indica*, then infested above ground on the first leaf with RWW (*Lissorhoptrus oryzophilus*) ‘adults’ at 26 DAG, and finally infested in the same root‐half with RWW ‘larvae’ at 30 DAG, leaving the other root‐half untreated, in fully crossed experiments in glasshouse. The biomasses of shoots, of untreated root‐half and of treated root‐half of the WT (a), *coi1‐18* (b), and *Eui1‐OX* plants (c) were measured and their values were analyzed by three‐way ANOVA or generalized linear model. Data are means ± SE: (a) *n *=* *8; (b, c) *n *=* *7–8. Significant *P*‐values of main factors effects are shown (E, endophyte; A, RWW adults; L, RWW larvae). For other *P*‐values see Supporting Information Table S6.

To test whether feeding by RWW larvae reduces root growth locally or systemically and whether the endophyte inhibits these effects on root growth, we measured the biomass of WT rice plants grown in a split‐root system. Adding RWW larvae to only one‐half of the root system resulted in no negative effects on the biomass of WT shoot and uninfested root‐half (Fig. 4a), but the biomass of the infested root‐half was significantly reduced by RWW larvae (Fig. 4a). No significant effects of RWW adults on root or shoot growth were detected, and significant interactions between the factors were also not detected (Table S6). However, a trend for an interaction between RWW adults and larvae was observed (Table S6). This trend suggests that prior adult herbivory may have worsened the local negative effect of conspecific larvae on the root biomass of WT plants (Fig. 4a). By contrast, endophyte inoculation increased the biomass of the inoculated root‐half and systemically increased the shoot biomass, and a trend suggested a slight systemic increase within roots of the uninoculated root‐half biomass of WT plants, compared with that of endophyte‐free WT plants (Fig. 4a; Table S6). Taken together, these results indicate that the effects of RWW larvae on root biomass in WT rice were confined primarily to the damaged sides of the root systems, but the effects were apparently worsened by previous leaf herbivory, whereas the endophyte had wider and positive systemic effects on the biomass of WT plants.

#### JA and GA antagonize systemic effects on plant growth

To test hypothesis H3, that JA and GA signaling mediates the effects of RWW and the endophyte on plant growth, we measured the biomass of JA‐insensitive *coi1‐18* and GA‐deficient *Eui1‐OX* rice mutants grown in split‐root systems. In contrast to the observed effects on WT plants, no effects of RWW adults or larvae were detected on any of the measured plant biomass components in the *coi1‐18* mutant (Fig. 4b; Table S6), suggesting that JA signaling is involved in mediating the reduction in growth of WT plants in response to RWW attack. By contrast, *coi1‐18* plants inoculated with the endophyte had larger shoot biomasses and root biomasses (both uninoculated root‐halves and inoculated root‐halves) compared with endophyte‐free *coi1‐18* plants (Fig. 4b). As these effects were stronger than those observed on WT plants, this indicates that JA signaling is a negative regulator of the plant growth‐promoting effects of *P. indica*. When the same fully crossed experiment using a split‐root system was conducted using the *Eui1‐OX* mutant, the endophyte had no detectable effects on any of the measured plant biomass components (Fig. 4c; Table S6), suggesting that the GA pathway was required for the plant growth‐promoting effects of *P. indica*. However, *Eui1‐OX* plants infested with RWW larvae had reduced shoot biomasses and reduced biomasses of infested root‐halves compared with uninfested *Eui1‐OX* plants (Fig. 4c). This suggests that shoot growth inhibition by RWW larvae is counteracted by GA in WT plants. Finally, RWW adults alone did not significantly affect any of the measured biomass components of *coi1‐18* and *Eui1‐OX* plants, and no interactions between factors were detected (Fig. 4b,c; Table S6). Overall, these results suggest that plant JA signaling promoted the detrimental effects of RWW and suppressed the beneficial effects of *P. indica*. By contrast, plant GA signaling suppressed the detrimental effects of RWW and promoted the beneficial effects of *P. indica*.

#### Susceptibility against RWW larvae in *P. indica*‐inoculated plants is reduced by JA signaling

The survival and weight of RWW larvae in WT plants were unaffected by the endophyte or by RWW adult feeding, and no interactions between factors were detected. The survival and weight of RWW larvae in WT plants were 33.79 ± 3.60% (*n *=* *32) and 7.73 ± 0.22 mg (*n *=* *29), respectively (mean ± SE; Table S7). The endophyte increased the survival of RWW larvae in *coi1‐18* plants from 24.22 ± 4.03 to 42.58 ± 4.61% compared with endophyte‐free *coi1‐18* plants (Table S7). RWW adults did not affect survival of RWW larvae in *coi1‐18* plants and no interaction between adults and the endophyte was detected. The weight of RWW larvae on *coi1‐18* plants was not affected by the endophyte or by the RWW adults, and no interaction between these factors was detected. The weight of RWW larvae on *coi1‐18* plants was 7.07 ± 0.24 mg (*n *=* *31). In *Eui1‐OX* plants, the survival of RWW larvae was not affected by the endophyte or by the RWW adults, and no interaction between these factors was detected. The survival of RWW larvae in *Eui1‐OX* plants was 22.07 ± 3.68% (*n *=* *32). Although the biomass of RWW larvae in *Eui1‐OX* plants was not significantly affected by any factor, and no interaction between factors was detected, we observed a trend for an effect by RWW adults (Table S7). This trend suggests that the biomass of RWW larvae may have increased slightly from 7.52 ± 0.32 in adult‐free *Eui1‐OX* plants to 8.97 ± 0.67 mg in *Eui1‐OX* plants infested with adults (*n *=* *12). Finally, no chlamydospores were observed in any rice genotypes at 38 d after fungal inoculation.

## Discussion

Mutualistic interactions between higher plants and microbes are increasingly recognized as important factors in terrestrial ecosystems. Positive effects of Sebacinalean root endophytes on plant growth, fitness, defense against pathogens, and tolerance to salt stress, as well as negative effects on resistance to leaf herbivory, have been documented (Varma *et al*., 1999; Barazani *et al*., 2005; Waller *et al*., 2005; Camehl *et al*., 2010), but their impact on root–herbivore interactions is unknown. Barazani *et al*. (2005) reported that *Sebacina vermifera* decreased the activity of proteinase inhibitors (PIs) in leaves of *Nicotiana attenuata* and consequently reduced leaf resistance to herbivory by *Manduca sexta*. This reduction resulted from endophyte‐inhibited ethylene (ET) signaling independent of JA signaling (Barazani *et al*., 2007). Our study demonstrates that plant–herbivore interactions are also affected by *P. indica*, a model endophyte with agronomic potential (Qiang *et al*., 2012). Contrary to Barazani *et al*. (2005), we observed an endophyte‐enhanced defense to herbivory manifested as increased root tolerance; that is, *P. indica*‐colonized plants infested with RWW larvae gained more shoot biomass, tillers, root biomass and total root length compared with plants infested with larvae without *P. indica*, but the root resistance measured as larval survival and growth was not affected by the endophyte. Therefore, Sebacinalean root endophytes, in addition to protecting plants against root and shoot pathogens and salt stress (Waller *et al*., 2005), can improve plant defense against root herbivores.

Although *P. indica* can affect ET signaling in roots (Camehl *et al*., 2010; Khatabi *et al*., 2012), ET does not regulate rice resistance or tolerance to root herbivory (Lu *et al*., 2015). Furthermore, we found no activity of PIs in submerged roots of rice (data not shown), confirming recent results (Lu *et al*., 2015). Thus, different signaling pathways for defense in above‐ and below‐ground tissues might explain why Sebacinalean‐mediated plant response to herbivory in our study contrasts with previous studies (Barazani *et al*., 2005, 2007). Consistent with the previous literature (Lu *et al*., 2015), we found only an attenuated twofold induction of JA in roots following root herbivory. This induction, however, was suppressed by *P. indica* without obvious effects on root resistance, that is, the survival and growth of the larvae were unchanged. JA is thought to be a master regulator of induced resistance to chewing herbivores (Howe & Jander, 2008), and AMF‐mediated increase of JA response can lead to improved leaf resistance to herbivory (Song *et al*., 2013), but the role of JA in root defense has been considered elusive (Erb *et al*., 2012a). For instance, the application of MeJA to rice roots reduced the survival of RWW larvae, but *hebiba* roots, which have a constitutive reduction in JA content, did not affect the larval survival or growth, whereas *as*LOX roots impaired in OPDA biosynthesis reduced larval growth as a result of lower nutritional quality of herbivore‐attacked roots, suggesting that 13‐lipoxygenase specifically improves root herbivore growth (Lu *et al*., 2015). Interestingly, in our study the concentrations of OPDA were not induced by herbivory in roots, in contrast to leaves. Furthermore, we found that larval performance in WT roots was similar to that in *coi1‐18* roots in the absence of *P. indica*, suggesting that herbivore‐induced JA signaling in roots is decoupled from root resistance. However, the *P. indica*‐enhanced larval survival in *coi1‐18* roots suggests that JA signaling may prevent the endophyte from increasing root susceptibility. Moreover, RWW larvae also induced JA‐Ile in roots, while *OsJAR1* expression was not affected. Even though JA signaling can regulate root resistance, plants may benefit from attenuating positive feedback loops of JA biosynthesis in roots to avoid nutritional enrichment that favors root herbivore growth and therefore could lead to greater injury. An attenuated induction of JA in roots therefore seems insufficient to affect the performance of RWW larvae, which could explain why *P. indica*‐mediated suppression of herbivore‐induced JA in roots did not affect root resistance. Furthermore, the interaction of *P. indica* with plants can be affected by JA signaling. Although the *coi1‐16* mutant of *Arabidopsis thaliana* did not affect *P. indica* colonization, the JA signaling *jin1‐1* and JA biosynthesis *jar1‐1* mutants caused a minor but significant reduction in *P. indica* colonization (Jacobs *et al*., 2011), while the exogenous application of MeJA to *N. attenuata* reduced the plant growth‐promoting effects of *P. indica* (Barazani *et al*., 2005). In our study, *P. indica* promoted the growth of *coi1‐18* more strongly than WT plants, which is additional evidence that JA signaling is a negative regulator of the plant growth‐promoting effects of *P. indica*.

A fundamental aspect that needs to be considered when studying JA signaling is that, in addition to plant defense, JA also regulates plant growth among other developmental processes (Hou *et al*., 2010; Yang *et al*., 2012; Riemann *et al*., 2015). In rice, root growth and elongation are reduced by exogenous application of MeJA (Staswick *et al*., 1992; Moons *et al*., 1997; Lu *et al*., 2015). In our study, we observed weak evidence for higher accumulation of jasmonates in roots when RWW adults and larvae were present and endophyte absent. This was associated with an additive negative effect on total root length. Stronger adverse effects on plant development of combined above‐ and below‐ground herbivory are often observed in nature (Zvereva & Kozlov, 2012). In our study, root inoculation with *P. indica* suppressed herbivore‐induced accumulation of JA in roots and enhanced plant tolerance to RWW attack. Furthermore, we detected only a local negative effect of RWW larvae on WT roots independent of *P. indica* inoculation, which tended to be worsened by leaf herbivory and was undetected in *coi1‐18* roots. Therefore, our results suggest that the negative effects of RWW herbivory on plant growth were mediated by induced JA signaling in roots. Intriguingly, the unaffected expression of *OsJAR1* in herbivore‐attacked roots suggests that *de novo* biosynthesis of JA pathway was inactive, while the reduced concentrations of JA in leaves following root herbivory suggest that herbivore‐induced JA in roots may be transported from the leaves. Zhang & Baldwin (1997) used [2‐^14^C]JA to demonstrate that direct transport of wound‐induced JA from leaves to roots accounts for the systemic increase of JA in roots of *Nicotiana sylvestris*. Therefore, a putative transport of JA from leaves to roots in our study could explain why prior herbivore‐induced JA in leaves may have contributed to greater JA accumulation in roots and therefore to stronger reduction of root development in response to root herbivory.

**Figure 5:**
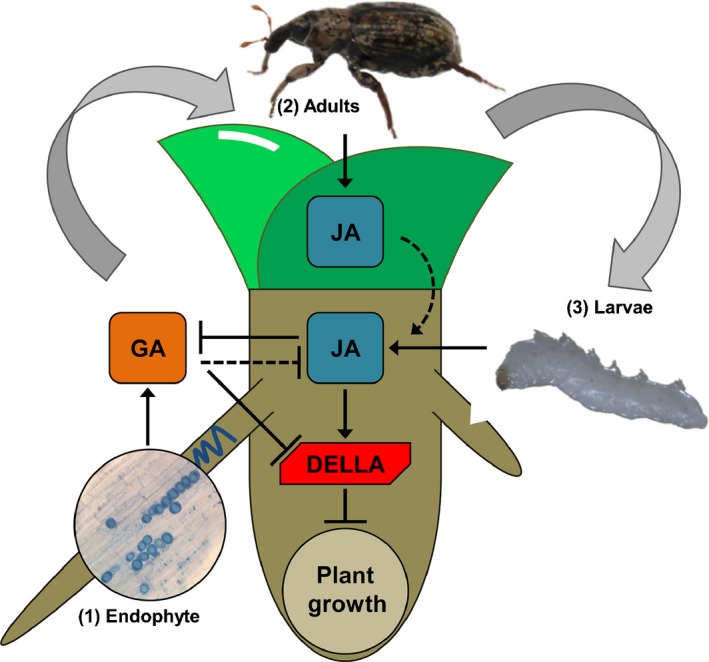
*S*chematic representation of endophyte‐induced plant tolerance to root herbivory. Rice (*Oryza sativa*) plants were first inoculated with the root endophyte *Piriformospora indica* (1), then infested above ground with rice water weevil (RWW;* Lissorhoptrus oryzophilus*) adults (2), and finally infested below ground with RWW larvae (3). The larvae induced jasmonic acid (JA) in roots, which was apparently enhanced by prior adult leaf herbivory, possibly through JA transport from leaves to roots. This could contribute to both the suppression of GA biosynthesis and the accumulation of DELLA and lead to plant growth inhibition. However, the previous endophyte colonization activates the GA biosynthetic pathway in roots to degrade DELLA and possibly to suppress JA accumulation. By disabling the JA mechanism for herbivore‐mediated plant growth inhibition, the endophyte induces plant tolerance to root herbivory. Arrows and blunt‐ended bars illustrate established positive and negative effects, respectively, and dashed lines indicate putative effects.

Mechanisms of JA‐mediated growth inhibition in above‐ground plant organs have been demonstrated for *A. thaliana*,* N. attenuata* and rice (Yang *et al*., 2012; Heinrich *et al*., 2013; Matschi *et al*., 2015). The current conception is that modulation of GA biosynthesis and JAZ interference with the interaction between DELLAs and growth‐promoting PIF transcription factors are two key mechanisms leading to JA‐mediated growth inhibition. In rice, overexpression of *EUI1* drastically reduces the concentrations of bioactive GAs (Zhu *et al*., 2006) and enhances the accumulation of DELLA (Luo *et al*., 2006), while the crossing of *Eui1‐OX* with *coi1‐18* plants showed that growth enhancement in *coi1‐18* plants depends on GA signaling (Yang *et al*., 2012). In our study, the stronger plant growth inhibition of *Eui1‐OX* plants following root herbivory suggests that the negative effects of RWW larvae on plant growth are counteracted by GA signaling in WT and *coi1‐18* plants. By contrast, *P. indica* colonization is known to up‐regulate several GA biosynthesis genes and down‐regulate a GA oxidase gene in *Hordeum vulgare*, while the GA‐deficient M117 and GA‐insensitive M121 mutants of *H. vulgare* and the GA‐deficient *ga1‐6* mutant of *A. thaliana* reduce *P. indica* colonization, whereas the quintuple‐DELLA mutant of *A. thaliana* can increase colonization (Schäfer *et al*., 2009; Jacobs *et al*., 2011). These prior results indicate that plant GA signaling is induced by, and required for, the mutualistic association with *P. indica*. It remains to be determined whether *P. indica* directly affects plant GA concentrations. Although it is unclear from our experiments whether the failure of *P. indica* to promote growth of *Eui1‐OX* mutant results from less fungal colonization or less induction of GA concentrations, our data confirm that GA signaling is induced by and required for the mutualistic association with *P. indica* in rice, as evidenced by the induction of *OsKS1* expression, stronger growth promotion of *coi1‐18* plants and failure to promote growth of *Eui1‐OX* plants. The involvement of GA as a negative regulator of JA signaling has been shown in *A. thaliana* (Hou *et al*., 2010; Wild *et al*., 2012), and it is tempting to speculate that *P. indica*‐elicited GA signaling might be also involved in the suppression of herbivore‐induced JA in rice roots. Furthermore, it is known that AMF can affect the interaction of plants with root herbivores through as‐yet‐uncharacterized mechanisms, and a role for mineral nutrition has been proposed (Gange *et al*., 1994; Gange, 2001; Cosme *et al*., 2011; Currie *et al*., 2011). To explore alternative explanations for the endophyte‐induced tolerance, we evaluated possible nutritional changes in rice plants and observed that the endophyte could not ameliorate the few nutritional deficits caused by root herbivory. Taken together, our results indicate that enhanced GA signaling and consequent suppression of JA signaling below ground comprise one mechanism by which an endophyte can induce plant tolerance to root herbivory (Fig. 5).

Although *P. indica* has not been found naturally in rice, and large‐scale experiments would be necessary to assess the robustness of the observed patterns in the field, our glasshouse study on wetland rice and recent studies on aerobic rice (Jogawat *et al*., 2013; Das *et al*., 2014) suggest that *P. indica* has the potential to be applied in rice production. Furthermore, while the role of fungal leaf endophytes in improving plant defense against above‐ground herbivory has been often documented, in particular for Clavicipitaceous endophytes and grasses (Rodriguez *et al*., 2009; Estrada *et al*., 2013), our study appears to be the first one reporting the impact of a root endophyte on plant defense to below‐ground herbivory. Moreover, we showed that endophyte‐enhanced plant defense to root herbivory resulted from induced plant tolerance, which adds to the growing evidence that induced compensatory regrowth may be an important defense strategy for roots to cope with the attack by herbivores (Poveda *et al*., 2010; Currie *et al*., 2011; Erb *et al*., 2012a; Robert *et al*., 2014). Finally, we demonstrate that GA signaling is one mechanism by which an endophyte can induce plant tolerance to root herbivory. This implies that GA is a putative signaling component of inducible compensatory regrowth against biotic stress. By showing how a fungal endophyte induces plant tolerance to root herbivory, our study illustrates a novel molecular mechanism underlying the integration of a beneficial microbe in the defense system of a higher plant.

## Author contributions

M.C., J.L., M.E., M.J.S., P.F. and S.W. designed the research. M.C. and J.L. performed the research. M.C. analyzed the data. M.C., M.E., M.J.S., P.F. and S.W. interpreted the data and wrote the manuscript.

## Supporting information

Please note: Wiley Blackwell are not responsible for the content or functionality of any supporting information supplied by the authors. Any queries (other than missing material) should be directed to the *New Phytologist* Central Office.

**Table S1** Primers used for qRT‐PCR
**Table S2** ANOVA results for Expt I on shoot biomass, tiller number and biomass, length and diameter of roots
**Table S3** Macronutrients and micronutrients in shoots and respective ANOVA or GLM results for Expt I
**Table S4** GLM results for Expt I on OPDA, JA and JA‐Ile in leaves and in roots
**Table S5** GLM results for Expt I on transcription abundance of OsJAR1 and OsKS1 in roots
**Table S6** ANOVA or GLM results for Expt II on biomass of shoots, untreated root‐half and treated root‐half
**Table S7** ANOVA or GLM results for Expt II on survival and growth of RWW larvae
