# Supplementary material for: A fungal endophyte helps plants to tolerate root herbivory through changes in gibberellin and jasmonate signaling
Source: New Phytol. 2016 Apr 6;211(3):1065–76. doi: 10.1111/nph.13957 (PMC5071772; doi:10.1111/nph.13957)
Supplement: Supplementary file 1 — Table S1 Primers used for qRT‐PCR Table S2 ANOVA results for Expt I on shoot biomass, tiller number and biomass, length and diameter of roots Table S3 Macronutrients and micronutrients in shoots and respective ANOVA or GLM results for Expt I Table S4 GLM results for Expt I on OPDA, JA and JA‐Ile in leaves and in roots Table S5 GLM results for Expt I on transcription abundance of OsJAR1 and OsKS1 in roots Table S6 ANOVA or GLM results for Expt II on biomass of shoots, untreated root‐half and treated root‐half Table S7 ANOVA or GLM results for Expt II on survival and growth of RWW larvae [file NPH-211-1065-s001.pdf]

New Phytologist

***New Phytologist* Supporting Information Tables S1–S7**

Article title: A fungal endophyte helps plants to tolerate root herbivory through changes in gibberellin and jasmonate signaling

Authors: Marco Cosme, Jing Lu, Matthias Erb, Michael Joseph Stout, Philipp Franken and Susanne Wurst

Article acceptance date: 25 February 2016

The following Supporting Information is available for this article:

Table S1 Primers used for qRT-PCR

Table S2 ANOVA results for Exp I on shoot biomass, tiller number and biomass, length and diameter of roots

Table S3 Macronutrients and micronutrients in shoots and respective ANOVA or GLM results for Exp I

Table S4 GLM results for Exp I on OPDA, JA and JA-Ile in leaves and in roots

Table S5 GLM results for Exp I on transcription level of OsJAR1 and OsKS1 in roots

Table S6 ANOVA or GLM results for Exp II on biomass of shoots, untreated root-half and treated root-half

Table S7 ANOVA or GLM results for Exp II on survival and growth of RWW larvae

New Phytologist

Table S1 Primers used for Quantitative real-time PCR analyses

Gene	RGAP LOCUS	Description	F-Primers (5'…3')	R-Primers (5'…3')
JAR1	LOC_Os05g50890	JA synthesis	AAGGTTTGTGAACCCATCAAACAGC	AATAATACTTTGCAGCACTTGTTACG
OsKS1	LOC_Os04g52230	GA synthesis	GACAAGGGACCAGCTCCAGACATTGGAG	CAGGAGCAGCAATCTGCTCATCCATGGC
OsACT	LOC_Os03g50885	Housekeeping	TGGACAGGTTATCACCATTGGT	CCGCAGCTTCCATTCCTATG

Table S2 Results of the three-way ANOVA for Exp I of the effects of the root endophyte *Piriformospora indica*, rice water weevil (RWW, *Lissorhoptrus oryzophilus*) adults, RWW larvae, and the interactions between their effects on shoot biomass FW, tiller number, root biomass FW, total root length and average root diameter of 58 d-old rice plants

Factors		Shoot FW		Tiller No		Root FW		Root length		Root diameter	
	df	*F*	*P*	*F*	*P*	*F*	*P*	*F*	*P*	*F*	*P*
Endophyte (E)	1	*2,984*	*0,090*	**5,906**	**0,018**	**5,177**	**0,027**	**9,056**	**0,004**	0,005	0,947
Adult (A)	1	0,970	0,329	0,024	0,877	0,238	0,628	1,555	0,218	0,046	0,831
Larva (L)	1	**9,954**	**0,003**	0,287	0,594	**1,966**	**0,167**	**16,060**	**<0,001**	**12,295**	**<0,001**
E x A	1	0,845	0,362	0,179	0,674	2,330	0,133	**7,882**	**0,007**	0,774	0,383
E x L	1	**5,371**	**0,024**	**9,534**	**0,003**	**7,001**	**0,011**	**21,696**	**<0,001**	0,194	0,661
A x L	1	0,097	0,756	0,389	0,535	0,126	0,724	1,128	0,293	0,389	0,535
E x A x L	1	0,017	0,897	0,055	0,816	0,621	0,434	**6,176**	**0,016**	1,315	0,256
Residuals	56										

Significant *P*-values (< 0.050) are given in bold, marginally significant *P*-values (< 0.100) are given in italic.

Table S3 Mean values of macronutrients (mg g^{-1} DW) and micronutrients (µg g^{-1} DW) in shoots of 58-d-old rice plants for Exp I as affected by the root endophyte *Piriformospora indica*, rice water weevil (RWW, *Lissorhoptrus oryzophilus*) adults, RWW larvae and their fully crossed combinations in comparison to the untreated control as well as the respective results of the three-way factorial ANOVA or GLM

**Macronutrients**	Nitrogen (N)			Phosphorus (P)			Potassium (K)			Calcium (Ca)			Magnesium (Mg)			Sulfur (S)		
mg g^{-1} DW	Mean		SE	Mean		SE	Mean		SE	Mean		SE	Mean		SE	Mean		SE
Control	29,05	±	1,75	5,447	±	0,082	51,22	±	0,80	4,863	±	0,141	2,543	±	0,082	2,775	±	0,079
Adult (A)	28,92	±	1,88	5,529	±	0,119	52,56	±	0,93	4,897	±	0,193	2,591	±	0,063	2,899	±	0,097
Larva (L)	28,67	±	3,00	4,459	±	0,281	46,08	±	3,36	6,078	±	0,552	2,394	±	0,085	2,980	±	0,198
AL	31,08	±	1,82	4,510	±	0,212	50,76	±	1,57	6,231	±	0,193	2,492	±	0,104	3,272	±	0,162
Endophyte (E)	29,89	±	1,73	5,514	±	0,098	52,79	±	1,53	4,786	±	0,094	2,439	±	0,072	2,781	±	0,115
EA	26,69	±	1,24	5,773	±	0,079	53,12	±	1,20	4,689	±	0,122	2,552	±	0,071	2,953	±	0,129
EL	27,90	±	1,29	4,856	±	0,066	50,54	±	0,77	5,641	±	0,208	2,545	±	0,056	2,966	±	0,066
EAL	28,51	±	1,45	4,827	±	0,058	49,28	±	1,15	5,297	±	0,109	2,396	±	0,066	2,863	±	0,127

Factors		GLM			GLM			GLM			GLM			ANOVA			ANOVA		
	df1	df2	*F*	*P*	df2	*F*	*P*	df2	*F*	*P*	df2	*F*	*P*	df2	*F*	*P*	df2	*F*	*P*
Endophyte	1	62	0,752	0,390	62	**5,205**	**0,026**	62	1,128	0,293	62	**6,743**	**0,012**	56	0,169	0,682	56	1,006	0,320
Adult	1	61	0,010	0,919	61	0,651	0,423	61	1,116	0,295	61	0,159	0,692	56	0,254	0,616	56	1,795	0,186
Larva	1	60	0,074	0,787	60	**64,938**	**<0,001**	60	**7,353**	**0,009**	60	**39,657**	**<0,001**	56	1,928	0,171	56	*3,452*	*0,068*
E x A	1	59	0,834	0,365	59	0,031	0,862	59	2,175	0,146	59	1,023	0,316	56	0,720	0,400	56	0,915	0,343
E x L	1	58	0,121	0,730	58	1,730	0,194	58	0,06	0,807	58	1,527	0,222	56	0,850	0,360	56	1,769	0,189
A x L	1	57	1,461	0,232	57	0,337	0,564	57	0,195	0,661	57	0,029	0,866	56	0,975	0,328	56	0,087	0,769
E x A x L	1	56	0,092	0,763	56	0,280	0,599	56	1,301	0,259	56	0,182	0,672	56	2,094	0,153	56	1,490	0,227

(Continued next page)

(Continuation of Table S3)

**Micronutrients**	Zinc (Zn)			Manganese (Mn)			Iron (Fe)			Copper (Cu)			Boron (B)			Molybdenum (Mo)		
µg g^{-1} DW	Mean		SE	Mean		SE	Mean		SE	Mean		SE	Mean		SE	Mean		SE
Control	117,2	±	4,45	382,1	±	10,47	286,9	±	38,26	13,78	±	0,67	9,626	±	0,287	1,953	±	0,082
Adult (A)	120,7	±	6,19	403,7	±	14,50	257,1	±	48,01	13,51	±	0,56	9,306	±	0,213	2,040	±	0,112
Larva (L)	130,5	±	12,04	316,8	±	40,49	261,3	±	20,36	17,96	±	4,87	11,633	±	0,876	2,249	±	0,080
AL	116,0	±	7,67	345,6	±	27,26	316,1	±	60,82	11,75	±	0,68	12,262	±	0,835	2,370	±	0,126
Endophyte (E)	118,6	±	3,91	401,7	±	6,75	243,2	±	18,89	13,14	±	0,80	9,678	±	0,272	2,072	±	0,097
EA	120,1	±	4,15	396,2	±	6,11	242,3	±	28,76	13,74	±	0,82	9,625	±	0,195	2,115	±	0,105
EL	118,7	±	4,38	365,5	±	10,12	264,0	±	28,18	13,57	±	0,26	11,306	±	0,576	2,154	±	0,057
EAL	111,5	±	4,96	360,8	±	10,68	317,7	±	41,16	12,98	±	0,57	11,055	±	0,853	2,105	±	0,077

Factors		ANOVA			GLM			ANOVA			GLM			GLM			ANOVA		
	df1	df2	*F*	*P*	df2	*F*	*P*	df2	*F*	*P*	df2	*F*	*P*	df2	*F*	*P*	df2	*F*	*P*
Endophyte	1	56	0,399	0,530	62	1,927	0,171	56	0,036	0,850	62	0,467	0,497	62	0,489	0,487	56	0,383	0,538
Adult	1	56	0,872	0,354	61	0,537	0,467	56	0,055	0,815	61	1,577	0,215	61	0,000	0,999	56	0,564	0,456
Larva	1	56	0,058	0,811	60	**12,659**	**<0,001**	56	1,889	0,175	60	0,156	0,695	60	**23,289**	**<0.001**	56	**6,829**	**0,012**
E x A	1	56	0,044	0,835	59	1,227	0,273	56	0,273	0,604	59	1,583	0,214	59	0,137	0,713	56	0,644	0,426
E x L	1	56	0,669	0,417	58	0,892	0,349	56	0,216	0,644	58	0,273	0,603	58	1,313	0,257	56	**4,313**	**0,042**
A x L	1	56	2,041	0,159	57	0,021	0,884	56	1,731	0,194	57	1,928	0,170	57	0,204	0,654	56	0,047	0,829
E x A x L	1	56	0,149	0,701	56	0,014	0,907	56	0,045	0,832	56	0,841	0,363	56	0,478	0,492	56	0,229	0,635

df1, degrees of freedom; df2, degrees of freedom of residuals. Significant *P*-values (< 0.050) are given in bold, marginally significant *P*-values (< 0.100) are given in italic.

Table S4 Results of the three-way GLM for Exp I of the effects of the root endophyte *Piriformospora indica*, rice water weevil (RWW, *Lissorhoptrus oryzophilus*) adults, RWW larvae and the interactions between their effects on 12-oxophytodienoic acid (OPDA), jasmonic acid (JA) and jasmonoyl-isoleucine (JA-Ile) in leaves and in roots of 58-d-old rice plants

Factors		Leaves							Roots						
			OPDA		JA		JA-Ile			OPDA		JA		JA-Ile	
	df1	df2	*F*	*P*	*F*	*P*	*F*	*P*	df2	*F*	*P*	*F*	*P*	*F*	*P*
Endophyte (E)	1	62	0,000	0,995	0,441	0,509	0,888	0,350	59	1,040	0,312	**6,048**	**0,017**	*3,155*	*0,081*
Adult (A)	1	61	**4,732**	**0,034**	**6,420**	**0,014**	**5,340**	**0,025**	58	0,336	0,565	1,524	0,222	1,513	0,224
Larva (L)	1	60	*3,483*	*0,067*	**4,554**	**0,037**	*3,274*	*0,076*	57	0,032	0,858	**7,385**	**0,009**	**14,864**	**<0,001**
E x A	1	59	0,237	0,628	1,662	0,203	1,424	0,238	56	0,069	0,794	0,829	0,367	0,984	0,326
E x L	1	58	0,594	0,444	0,047	0,829	0,609	0,438	55	2,079	0,155	**4,133**	**0,047**	1,657	0,204
A x L	1	57	0,047	0,830	2,015	0,161	1,368	0,247	54	0,196	0,660	0,588	0,447	0,024	0,877
E x A x L	1	56	0,608	0,439	0,533	0,469	0,348	0,558	53	0,291	0,592	0,674	0,415	0,909	0,345

df1, degrees of freedom; df2, degrees of freedom of residuals. Significant *P*-values (< 0.050) are given in bold, marginally significant *P*-values (< 0.100) are given in italic.

New Phytologist

Table S5 Results of the three-way GLM for Exp I of the effects of the root endophyte *Piriformospora indica*, rice water weevil (RWW, *Lissorhoptrus oryzophilus*) adults, RWW larvae and the interactions between their effects on the relative transcription levels of *OsJAR1* and *OsKS1* genes in roots of 58 d-old rice plants

Factors			*OsJAR1*		*OsKS1*	
	df1	df2	*F*	*P*	*F*	*P*
Endophyte (E)	1	59	0,574	0,452	**6,037**	**0,017**
Adult (A)	1	58	0,166	0,685	0,000	0,993
Larva (L)	1	57	0,138	0,711	**48,198**	**<0,001**
E x A	1	56	0,013	0,910	1,723	0,195
E x L	1	55	*3,475*	*0,068*	0,768	0,385
A x L	1	54	2,436	0,125	0,109	0,743
E x A x L	1	53	0,678	0,414	*3,420*	*0,070*

df1, degrees of freedom; df2, degrees of freedom of residuals. Significant *P*-values (< 0.050) are given in bold, marginally significant *P*-values (< 0.100) are given in italic.

Table S6 Results of the three-way ANOVA or GLM for Exp II of the effects of the root endophyte *Piriformospora indica*, rice water weevil (RWW, *Lissorhoptrus oryzophilus*) adults, RWW larvae and the interactions between their effects on the FW of shoots, untreated root-half, and treated root-half of 58-d-old WT, *coi1-18*, and *Eui1-OX* plant lines.

WT		Shoot			Untreated root-half			Treated root-half		
		ANOVA			GLM			GLM		
Factors	df1	df2	*F*	*P*	df2	*F*	*P*	df2	*F*	*P*
Endophyte (E)	1	56	**4,738**	**0,034**	62	*3,290*	*0,075*	62	**5,223**	**0,026**
Adult (A)	1	56	0,197	0,659	61	0,486	0,488	61	0,354	0,554
Larva (L)	1	56	0,620	0,434	60	0,217	0,643	60	**4,030**	**0,049**
E x A	1	56	0,014	0,906	59	0,003	0,959	59	0,020	0,887
E x L	1	56	0,219	0,642	58	1,178	0,282	58	0,720	0,400
A x L	1	56	1,842	0,180	57	0,810	0,372	57	*3,234*	*0,078*
E x A x L	1	56	0,179	0,674	56	0,220	0,641	56	0,950	0,334

coi1-18		Shoot			Untreated root-half			Treated root-half		
		GLM			GLM			GLM		
Factors	df1	df2	*F*	*P*	df2	*F*	*P*	df2	*F*	*P*
Endophyte (E)	1	61	**16,305**	**<0,001**	61	**19,430**	**<0,001**	61	**17,243**	**<0,001**
Adult (A)	1	60	2,295	0,136	60	1,870	0,177	60	1,609	0,210
Larva (L)	1	59	0,140	0,710	59	0,028	0,869	59	0,666	0,418
E x A	1	58	0,263	0,610	58	0,352	0,556	58	0,791	0,378
E x L	1	57	0,558	0,458	57	1,121	0,294	57	0,019	0,892
A x L	1	56	0,242	0,625	56	0,524	0,472	56	0,720	0,400
E x A x L	1	55	0,000	0,997	55	0,979	0,327	55	0,323	0,572

Eui1-OX		Shoot			Untreated root-half			Treated root-half		
		GLM			GLM			GLM		
Factors	df1	df2	*F*	*P*	df2	*F*	*P*	df2	*F*	*P*
Endophyte (E)	1	57	2,155	0,148	57	2,599	0,113	57	0,581	0,449
Adult (A)	1	56	0,975	0,328	56	0,861	0,358	56	0,578	0,451
Larva (L)	1	55	**5,612**	**0,022**	55	2,505	0,120	55	**5,789**	**0,020**
E x A	1	54	0,703	0,406	54	1,018	0,318	54	0,480	0,492
E x L	1	53	0,212	0,647	53	0,000	0,999	53	0,002	0,963
A x L	1	52	0,279	0,600	52	1,267	0,266	52	0,567	0,455
E x A x L	1	51	1,203	0,278	51	0,226	0,637	51	0,252	0,618

df1, degrees of freedom; df2, degrees of freedom of residuals. Significant *P*-values (< 0.050) are given in bold, marginally significant *P*-values (< 0.100) are given in italic.

New Phytologist

Table S7 Results of the two-way ANOVA or GLM for Exp II of the effects of the root endophyte *Piriformospora indica*, rice water weevil (RWW, *Lissorhoptrus oryzophilus*) adults, and the interaction between their effects on survival and growth of RWW larvae 28 d after neonate infestation in roots of 58-d-old WT, *coi1-18* or *Eui1-OX* plant lines

		WT						*coi1-18*						*Eui1-OX*					
		Survival			Growth			Survival			Growth			Survival			Growth		
		GLM			GLM			ANOVA			GLM			ANOVA			GLM		
Factors	df1	df2	*F*	*P*	df2	*F*	*P*	df2	*F*	*P*	df2	*F*	*P*	df2	*F*	*P*	df2	*F*	*P*
Endophyte (E)	1	30	0,131	0,721	27	1,085	0,308	28	**9,048**	**0,006**	29	0,083	0,775	28	0,688	0,414	22	0,161	0,693
Adults (A)	1	29	0,066	0,799	26	0,071	0,792	28	0,692	0,412	28	0,072	0,791	28	0,199	0,659	21	*4,122*	*0,056*
E x A	1	28	0,237	0,630	25	0,035	0,854	28	1,479	0,234	27	0,005	0,944	28	0,066	0,799	20	0,016	0,900

WT, wild type; df1, degrees of freedom; df2, degrees of freedom of residuals. Significant *P*-values (< 0.050) are given in bold, marginally significant *P*-values (< 0.100) are given in italic.
